# Transfer of knowledge in international cooperation: the Farmanguinhos – SMM case

**DOI:** 10.11606/S1518-8787.2017051006249

**Published:** 2017-11-13

**Authors:** Samuel Araujo Gomes da Silva, Roberto Gonzalez Duarte, José Márcio de Castro

**Affiliations:** I Universidade Federal de Minas Gerais. Faculdade de Ciências Econômicas. Centro de Desenvolvimento e Planejamento Regional. Programa de Pós-Graduação em Demografia. Belo Horizonte, MG, Brasil; II Universidade Federal de Minas Gerais. Faculdade de Ciências Econômicas. Departamento de Administração. Belo Horizonte, MG, Brasil; III Pontifícia Universidade Católica de Minas Gerais. Instituto de Ciências Econômicas e Gerenciais. Programa de Pós-Graduação em Administração. Belo Horizonte, MG, Brasil

**Keywords:** Knowledge Management, Technology Transfer, International Educational, Exchange International Cooperation, Health Human Resource Training, Gestão do Conhecimento, Transferência de Tecnologia, Intercâmbio Educacional Internacional, Cooperação Internacional, Capacitação de Recursos Humanos em Saúde

## Abstract

**OBJECTIVE:**

To analyze the influence of four mechanisms of knowledge transfer (training, technical visits, expatriation, and standard operating procedures) on the different dimensions (potential and realized) of absorptive capacity in international technical cooperation.

**METHODS:**

We examine the case of implementation of the *Sociedade Moçambicana de Medicamentos*. Data have been collected using semi-structured interviews (applied to 21 professionals of the *Sociedade Moçambicana de Medicamentos*, Farmanguinhos, FIOCRUZ, and Itamaraty) and official documents. The data of the interviews have been submitted to content analysis, using the software NVivo.

**RESULTS:**

Training and technical visits directly influenced the acquisition and, partly, the assimilation of knowledge. Expatriation contributed with the transformation of this knowledge from the development and refinement of operational routines. Finally, the definition of standard operating procedures allowed the Mozambican technicians to be the actors of the transformation of the knowledge previously acquired and assimilated and, at the same time, it laid the foundations for a future exploration of the knowledge.

**CONCLUSIONS:**

Training and technical visits mainly influence the potential absorptive capacity, while expatriation and standard operating procedures most directly affect the realized absorptive capacity.

## RESUMO

**OBJETIVO:**

Analisar a influência de quatro mecanismos de transferência de conhecimento (treinamentos, visitas técnicas, expatriação e procedimentos operacionais padrão) sobre as diferentes dimensões (potencial e realizada) da capacidade absortiva na cooperação técnica internacional.

**MÉTODOS:**

Examina-se o caso da implementação da Sociedade Moçambicana de Medicamentos. Os dados foram coletados por meio de entrevistas semiestruturadas (aplicadas a 21 profissionais da Sociedade Moçambicana de Medicamentos, Farmanguinhos, Fiocruz e Itamaraty) e de documentos oficiais. Os dados das entrevistas foram submetidos à análise de conteúdo, com uso do *software* NVivo.

**RESULTADOS:**

Os treinamentos e as visitas técnicas influenciaram diretamente a aquisição e, parcialmente, a assimilação do conhecimento. A expatriação contribuiu para a transformação desse conhecimento, por meio do desenvolvimento e refinamento das rotinas operacionais. Por fim, a definição dos procedimentos operacionais padrão permitiu que os técnicos moçambicanos fossem os atores da transformação do conhecimento adquirido e assimilado previamente e, ao mesmo tempo, criou as bases para uma futura exploração do conhecimento.

**CONCLUSÕES:**

Os treinamentos e as visitas técnicas influenciam, principalmente, a capacidade absortiva potencial, enquanto a expatriação e os procedimentos operacionais padrão impactam mais diretamente a capacidade absortiva realizada.

## INTRODUCTION

The process of knowledge transfer is the process of sharing tacit and explicit knowledge between two agents^12^, and its success is associated with the ability to recreate transferred knowledge in a new context^17^. The process of knowledge transfer is effective and complete^11^ if knowledge is resignified for the understanding of the individuals of the recipient organization and internalized by them^17^. These three actions – resignification, understanding, and internalization of the transferred knowledge – depend largely on the absorptive capacity of the recipient organization^6^.

The absorptive capacity is subdivided into two dimensions: potential absorptive capacity and realized absorptive capacity. The first one comprises the skills of acquisition (identifying and acquiring critical external knowledge for the operation of the organization) and assimilation (organizing routines and processes that allow the organization to analyze, process, interpret, and understand the knowledge obtained from external sources; that is, the process that the externally acquired knowledge is distributed throughout the recipient organization). The second one involves the skills of transformation (developing and refining routines that facilitate the combination of existing and previously acquired knowledge) and exploration (refining, extending, and leveraging capabilities or creating new ones by incorporating the knowledge acquired and transformed into operations)^19^.

Although the two dimensions are interrelated and interdependent, external knowledge can be acquired and assimilated without being transformed and explored; in this case, knowledge transfer is incomplete and non-effective. Similarly, if the potential absorptive capacity is low, knowledge can be acquired or assimilated, but only partially, which affects the realized absorptive capacity. In this case, the process of knowledge transfer is also incomplete and non-effective. The potential absorptive capacity is thus *sine qua non*, though not enough, for a complete process of knowledge transfer. Although the literature broadly discusses the transfer of intra- and interorganizational knowledge^7^ and, in particular, the mechanisms for its operationalization^12^ and the role of absorptive capacity in this process^6^, we still do not know much about this phenomenon in the context of international technical cooperations.

In recent years, Brazil has actively participated in cooperative actions with other developing countries, especially in Latin America and Africa^a^. In some African countries, for example, some common obstacles have been identified in their public health systems: considerable shortages of skilled personnel, excessive dependence on foreign physicians combined with the emigration of more qualified professionals, low capacity for the formation of human health resources, insufficient remuneration of health professionals, absence of an information system on human resources in the health sector, weak management capacity, and low financial potential for a rapid expansion of human resources in health. In view of these structural problems, Brazil has adopted a structural cooperation model, whose objective is capacity building (training of human resources, organizational strengthening, and institutional development)^1^ ^,^ ^4^ ^,^ ^9^ ^,^ ^14^, in which knowledge transfer is the main means of operationalization^b^ ^,^ ^c^.

Given the growth of South-South technical cooperation, that is, between developing countries, in the health sector, this study aimed to describe and analyze the interrelationships between the mechanisms of knowledge transfer, the absorptive capacity of the recipient unit, and the results of the process – capacity building^d^. We highlight that this capacity building depends on the mechanisms chosen for knowledge transfer and how they are used to leverage the potential and realized absorptive capacity. The appropriate choice and combination of mechanisms can ensure the effectiveness of the transfer between source and recipient. This issue is critical in horizontal South-South technical cooperation, as the recipient organization is usually in a context of low absorptive capacity, which may negatively influence the process and outcome of the transfer. In order to discuss these interrelations in this context, we examine the case of the implementation of the Sociedade Moçambicana de Medicamentos (SMM), the first public pharmaceutical factory in a sub-Saharan African country^5^, with the technical support of Farmanguinhos, a drug industry of the Fundação Osvaldo Cruz (FIOCRUZ).

## METHODS

In order to carry out this research, we chose the case study^18^ of qualitative nature^8^ for the intensive examination – both in amplitude and in depth – of a study unit^10^. Data were collected using semi-structured interviews^16^ and document analysis, such as the technical-economic viability study carried out by Farmanguinhos.

The interview script was structured around two main variables: mechanisms of knowledge transfer and dimensions of the absorptive capacity. Regarding the first one, four mechanisms were investigated: (i) training, (ii) expatriation, (iii) technical visits, and (iv) Standard Operating Procedures (SOP)^15^. In relation to the second one, we analyzed the skills of acquisition, assimilation, processing, and exploration.

We carried out 29 interviews with eight Brazilians, eleven Mozambicans, one Portuguese, and one Zimbabwean, all directly involved in the implementation process of the SMM (some professionals were interviewed more than once). The interviews were held in Farmanguinhos, Rio de Janeiro, Brazil, on two occasions (March-April and October 2013), and SMM, Maputo, Mozambique, also on two occasions (May and October-November 2013). Of the 29 interviews, 28 were recorded, as one interviewee objected to the storage of the audio but authorized the use of annotations during the interview for the analysis of the data. Interviewees are identified as follows: number of the interviewee and, when interviewed more than once, interview number (e.g., interviewee 3:2 refers to the information obtained in the second interview of interviewee 3). The audios of the interviews, which lasted between 25 and 112 minutes, were transcribed literally. Transcripts resulted in approximately 300 pages of text. This first treatment of the data favored the manipulation of the material^3^ and a first reading, in order to understand the data in their fullness.

Then, using the software NVivo, we analyzed the content^2^ of the interviews for the codification, categorization, and classification of the units of text. To this end, we performed: (i) classification of the data in the defined categories (mechanisms of knowledge transfer and dimensions of the absorptive capacity), (ii) cluster analysis to group interviewees and categories according to the similarity of words and their frequency, and (ii) word cloud to see the most used terms and their frequency. These analyses helped us investigate similarities in the information, the alignment between the declarations of the various sources, and the identification of the most used terms and words in the reports.

Regarding the analysis of the documents, the main source was the study of technical and financial feasibility, carried out by FIOCRUZ, to subsidize the implementation of the SMM. The study contains from the diagnosis of the situation of the HIV epidemic in Mozambique up to the identification of the profile and the amount of personnel required to operate the factory. The analysis of the report helped us understand the socioeconomic conditions of the country before the installation of the factory and how these conditions could affect the knowledge transfer between Farmanguinhos and SMM.

## RESULTS

The Agência Brasileira de Cooperação, in partnership with other agencies and ministries, currently has projects in approximately 50 countries in various areas, such as collective health, agriculture, and renewable energy^e^. The Brazilian international technical cooperation, especially in Portuguese-speaking countries, is still concentrated in the health sector, especially in the fight against HIV/AIDS^13^.

The project that resulted in the implementation of SMM was mentioned for the first time in 2003 by the then president of Brazil, Luís Inácio Lula da Silva, on an official trip to Mozambique. Between 2005 and 2007, technical and financial feasibility studies were developed. Once feasibility was verified, the phase of project execution started, which was financed by the Brazilian and Mozambican governments. At that time, diplomatic agreements were signed regarding the cooperation between the two countries and professionals who would work on the design and implementation of the factory were contracted (interviews 1, 3).

Considering the purpose of the cooperation action (capacity building), the complexity of the pharmaceutical manufacturing operation, and the small number of professionals in the pharmaceutical production area in Mozambique, one of the priorities in the first phase of the project implementation was to transfer knowledge. After evaluating the qualifications and previous experience of the newly hired professionals and comparing them with the skills required for the implementation and operation of the factory, a training and qualification plan was elaborated, and the most appropriate training needs and mechanisms of transfer were defined: training, technical visits, expatriation, and SOP (interviews 1, 3).

Brazilian missions were frequently used to discuss the operational details of the project in the initial phase of the implementation of the SMM; thus, Farmanguinhos professionals were invited to offer short courses – between 10 and 12 hours (interviews 3, 9:1, 12) – for the technicians of the former *Final Farmacêutica* (a private serum factory that was bought by the Mozambican government to install the SMM) and the Ministry of Health designated to work in the operation. The first trainings aimed to present basic notions about the pharmaceutical industry – from structure to drug manufacturing practices. The pharmaceutical manufacturing process is highly standardized and SOP are required for the accreditation of a factory by control agencies, such as the Brazilian Health Surveillance Agency and the Food and Drug Administration; therefore, the training on manufacturing routines was the basis for the technicians who would work in the factory. Furthermore, equipment manufacturers also offered short-term training. As the machines were installed, training was given on their operation and then practical tests were applied to see if the technicians had effectively understood the operation of the equipment (interviewees 10:2, 13, 15, 17, 20). According to one interviewee, “while setting up this factory, we were also following the steps of installation of the machines. It was a type of class [...] that combined theory and practice” (interview 14). Another type of training was the graduate training, justified by the need for a continuous learning process, since the pharmaceutical industry is dynamic and intense in innovations. However, until the end of the data collection, the only person who had started graduate studies after hiring was the executive director, who was finishing a master’s degree in business management in Portugal (interviewees 3, 4:2; 4:3, 12). The main objective of the training was to increase the potential absorptive capacity, mainly knowledge acquisition.

Although the trainings were necessary for knowledge transfer, they alone would be insufficient to leverage the other dimensions of the potential and realized absorptive capacities, specially assimilation and transformation. Technical visits and expatriation focused on these two dimensions. Nineteen Mozambicans from both management and production were sent to Farmanguinhos, where they spent between 20 and 30 days. The purpose was to allow technicians to follow the daily routine of productive activities, to get closer to Brazilian professionals with experience in the tasks in the areas they were being prepared to work, and to transfer the knowledge acquired in Farmanguinhos to colleagues in SMM (interviewees 1, 3, 4:1, 4:2, 9:1, 10:1, 12).

In order to evaluate the knowledge acquired during the technical visits, short tests were elaborated that were the basis for the construction of the implementation plan of this knowledge in the context of the factory. This plan detailed how the newly learned knowledge should be applied and, if so, how it would be appropriate to the reality of the SMM (interviews 5:3, 7:2, 12, 13, 21). When the Mozambican technicians effectively returned to Maputo with their implementation plan in hand, a Brazilian technician accompanied them to help them put the plan into practice, continuing the process of assimilation of the knowledge acquired. The Brazilians spent approximately 15 days in Maputo, interspersed with periods of two months in Brazil. If, in the absence of these professionals, problems arose, other Brazilians, who worked full-time in the factory, were called upon to solve those (interviews 8, 12).

The training and technical visits contributed with the acquisition of certain knowledge, but the assimilation demanded a more exhaustive and intensive follow-up by Brazilian professionals. Three expatriates, with extensive experience in the pharmaceutical industry, followed the implementation process of the factory – from the installation of machines to their effective operation, going through the definition of production routines and processes. Two worked in production and quality, the most sensitive areas of the factory. The quality area covered both the control and quality assurance sectors. They assisted the technicians of the SMM in the assimilation, that is, in the contextualization of the knowledge acquired, and in the application of this knowledge aiming at the creation of drug manufacturing routines for SMM (interviews 3, 10:1, 12). The other expatriate supported the Mozambicans in the management of the factory, acting as an advisor, that is, when there were problems, he helped to design the most appropriate solutions to solve them. In addition to working directly for the transfer of technical and managerial knowledge, these expatriates were also a link between SMM and Farmanguinhos, contributing with the flow of knowledge between the two parties.

By sending these professionals to Maputo, they could get closer to Mozambicans, allowing them to understand better not only the specificities of the SMM but also the – social, economic, and political – reality of the country. This broader understanding of the context consequently helped them to understand the most appropriate ways to transfer and apply the knowledge about drug manufacturing and management to the SMM. We highlight that the expatriates worked together with the Mozambican technicians to create solutions to the specific problems of the factory (interviews 6:1, 19, 21). Unlike the training and technical visits that directly influenced the acquisition and partially the assimilation of knowledge, expatriation affected the realized absorptive capacity, that is, it contributed with the transformation of this knowledge from the development and refinement of operational routines.

Finally, instead of simply transferring the SOP, the good manufacturing practices, and the technical data records of Farmanguinhos, the documents leveraged the transformation while creating the basis for a future exploration of knowledge. The direct participation of Mozambican technicians in the process of adaptation and recreation of SOP in the local reality was thus favored. The technicians of the area described the procedures, which were then sent to the quality sector to see if the text was clear, accessible, and in line with international standards. If so, the document was approved and then the technicians would follow the procedure they had described. Finally, evaluations of the implementation of the process as described in the SOP were carried out and, if necessary, adjustments were suggested (interviews 5:3, 18). The definition of the procedures based on the reality of the SMM (interview 7:2) allowed Mozambican technicians to be the actors of the transformation of the previously acquired and assimilated knowledge. More than adapting the SOP, the professionals of the SMM elaborated them themselves, ensuring the achievement of one of the main objectives of the implementation project of the factory: capacity building and, consequently, sustainability of the initiative.

Box 1 summarizes the knowledge transfer mechanisms used, the target audience, and the objectives of the process.

**Box 1:** Mechanisms of knowledge transfer used in the implementation of the SMM.

Mechanism	Target audience	Objective
Trainings (Short courses, courses on equipment, and graduation)	Professionals of the old serum factory Technicians of the Ministry of Health Main manager	Presentation of the basics about the structure of a drug factory, drug manufacturing practices, and manufacturing behaviors. Operation of machines and equipment. Master’s in business management.
Technical visits (Mozambicans in Farmanguinhos and Brazilian in SMM)	Mozambican technicians Brazilian technicians	Monitoring of the daily activities of drug production. Approximation between the Mozambican and Brazilian professionals. Dissemination of knowledge. Application of the knowledge in SMM.
Expatriation	Brazilian technicians in the areas of production and quality Brazilian technician in the area of management of drug industries	Help in the internalization of the knowledge acquired. Monitoring of the (productive and management) processes of factory implementation. Creation of drug manufacturing routines, together with technicians of the SMM. Consultancy for the design of solutions.
Standard Operating Procedures (SOP), Good Manufacturing Practices, and Technical Records	Mozambican technicians	Access to standard procedures, good pharmaceutical practices, and technical records, which provide insights into the process of drug production. Adaptation of standard procedures, good pharmaceutical practices, and technical records, with the direct participation of local employees.

SMM: *Sociedade Moçambicana de Medicamentos*

## DISCUSSION

The implementation of the SMM showed the close interrelationship between the mechanisms of knowledge transfer and the different dimensions of the absorptive capacity and the complementarity between these mechanisms for the effectiveness of the transfer process.

All mechanisms directly or indirectly affect, to a greater or lesser extent, both the potential and realized absorptive capacity, but each of the mechanisms analyzed tends to be more relevant to a specific dimension. Initial training provided a knowledge base to the employees of the SMM who had no experience or involvement in drug production (i.e., prior knowledge of the production process). Afterwards, several of these professionals were sent to Brazil to experience the routine of a fully functioning factory. Technical visits provided an opportunity to gain knowledge. Upon returning to Maputo, the work of the technicians was accompanied by Brazilian expatriates who supervised the application of the knowledge acquired in the previous phases, thus materializing assimilation. Finally, the Mozambican technicians, with the support of the expatriates, elaborated operational procedures, such as SOP, good manufacturing practices, and technical records. This elaboration allowed the application and, mainly, the transformation of knowledge.

The influence and relevance of each mechanism were confirmed by cluster analysis, which groups words by similarity, using Pearson correlation coefficient as a metric. In the result provided by NVivo, the first categories grouped were: (i) technical visits and assimilation, (ii) expatriation and processing, and (iii) SOP and exploration. As evidenced by the results of NVivo, the training in Farmanguinhos contributed with the acquisition, but it also started the process of assimilation, that is, analysis, processing, interpretation, and understanding of information shared at this and in the previous phases. Expatriation, on the other hand, was important for the consolidation of assimilation, but above all, it was decisive for initiating the transformation (i.e., contextualization) of the knowledge acquired. Similarly, technicians transformed their prior knowledge with the development of fundamental standards and routines in an industry that is based on accurate and detailed production practices. In addition, the development of SOP, with the support of expatriates, created the necessary basis for a possible exploration of the knowledge in the future. Thus, drug production standards are the central axis around which the process of knowledge transfer is organized. In addition, if the mechanisms individually have different roles in the different steps of the process of knowledge transfer, they are jointly and integrally responsible for the effectiveness of the process.

Box 2 illustrates the influence of each mechanism for the different dimensions of the absorptive capacity.

**Box 2:** Influence of the mechanisms of knowledge transfer for the dimensions of absorptive capacity.

Mechanism		Potential absorptive capacity		Realized absorptive capacity	
	
		Acquisition	Assimilation	Transformation	Exploration
Trainings	Short courses	↑	↑	→	→
	Graduate course	↑	→	→	→
	Training by equipment manufacturers	↑	↑	→	→
Technical visits	Visits of Brazilian technicians to Farmanguinhos Visits of Brazilian technicians to SMM	↑	↑	→	→
Expatriation	Support to the application of knowledge in the construction of routines in the areas of quality and production Support for solving management problems	→	↑	↑	→
Manuals/SOP	Preparation of SOP, GMP, and TR	→	→	↑	→

→ Moderately influence the size of the absorptive capacity.

↑ Die size of the absorptive capacity.

The absorption of knowledge and its exploration are naturally complex actions, which depend on the existence of circumstances and mechanisms that favor it. As South-South technical cooperation actions are developed in countries with low absorptive capacity, the transformation and exploration of technical knowledge, sometimes of highly complexity, can be difficult. In the case of SMM, there is still no evidence of this full and effective exploration of knowledge, since the analysis was limited to the implementation period of the factory.

The research has implications for managers involved in horizontal technical cooperation actions. The transfer of knowledge between developing countries (in particular with low absorptive capacity available) requires, in a first moment, mechanisms that favor the acquisition and the assimilation of knowledge. However, since capacity building also requires the development of the transformation and exploration capacities, the choice of mechanisms that leverage the realized absorptive capacity is a concern for these managers, since these capacities are determinant for the sustainability of the action. Moreover, as discussed above, rather than choice, the proper integration between the mechanisms is responsible for the potential of the realized absorptive capacity.

A case study notoriously has its limitations, the main one being the impossibility of generalizations. This research focused on the analysis of knowledge transfer in the technical cooperation among developing countries in the health area. Possibly, the mechanisms of transfer and the dimensions of the absorptive capacity are differently interrelated in technical cooperations in other areas. Another limitation refers to the specific context analyzed in this research: shortage of skilled labor in the pharmaceutical area. Other contexts with more structured knowledge bases probably require other arrangements for knowledge transfer. Considering these limitations, we suggest that future research studies should analyze the process in other areas of cooperation, such as education, agriculture, and public management, in order to analyze the role of the knowledge base of the recipient country in choosing the mechanisms used in the process of knowledge transfer.

## Footnotes

a Ministério das Relações Exteriores (BR), Agência Brasileira de Cooperação. Cooperação técnica. Brasília (DF); c2012 [cited 2012 May 26]. Available from: http://www.abc.gov.br

b Fundação Oswaldo Cruz. Cooperação técnica entre países em desenvolvimento – Brasil/Moçambique: sumário executivo: estudo de viabilidade técnico-econômico para instalação da fábrica de medicamentos em Moçambique para produção de anti-retrovirais e outros. Rio de Janeiro: Fiocruz; 2007.

c Fundação Oswaldo Cruz. Cooperação Técnica Internacional Brasil-Moçambique: projeto de instalação da fábrica de anti-retrovirais e outros medicamentos. Rio de Janeiro: Fiocruz; 2012.

d Fundação Oswaldo Cruz (Fiocruz). Cooperação Técnica entre Países em Desenvolvimento Brasil/Moçambique: sumário executivo: estudo de viabilidade técnico-econômico para instalação da fábrica de medicamentos em Moçambique para produção de anti-retrovirais e outros. Rio de Janeiro: Fiocruz; 2007.

e Ministério das Relações Exteriores (Brasil), Agência Brasileira de Cooperação. Cooperação técnica. Brasília (DF); c2012 [cited 2012 May 26]. Available from: http://www.abc.gov.br
